# Immunoexpression of P63 and SOX2 in triple-negative breast cancers, Indonesia

**DOI:** 10.12688/f1000research.12671.2

**Published:** 2018-01-08

**Authors:** Reno K Kamarlis, Muhammad ND Lubis, Bethy S Hernowo, Azmi S Kar

**Affiliations:** 1 Department of Pathology Anatomy, Dr. Zainoel Abidin General Hospital, Banda Aceh, Indonesia; 2 Department of Pathology Anatomy, School of Medicine, Syiah Kuala University, Banda Aceh, Indonesia; 3 Department of Pathology Anatomy, Medical Faculty, Sumatera Utara University, Medan, Indonesia; 4 Department of Pathology Anatomy, Medical Faculty, Padjajaran University, Bandung, Indonesia; 5 Department of Internal Medicine, Medical Faculty, Sumatera Utara University, Medan, Indonesia

**Keywords:** breast cancer, breast cancer marker, P63, SOX2, triple negative breast cancers

## Abstract

**Background**: Using immunohistochemical stains to target specific breast cancer markers has become indispensable for evaluation of small diagnostic tissue specimens, and therefore novel marker cocktails for specific breast cancers are required. This study was conducted to assess the immunoexpression of P63 and SOX2 in triple negative breast cancer (TNBC), and to evaluate the predictive diagnostic value of these markers for specific types of TNBC.

**Methods**: Histological slides and paraffin blocks of TNBC cases were collected from Dr. Hasan Sadikin Hospital, Bandung, Indonesia from 5-years period (2011-2015). Each histological slide was subjected to immunohistochemical staining for P63 (nucleus and cytoplasm) and SOX2 (nucleus), with specific primer antibodies. Immunoexpression of P63 and SOX2 was evaluated using immunoreactivity scoring. Associations between P63 and SOX2 immunoexpression and TNBC types were assessed using Mann Whitney tests. In addition, the predictive diagnostic values of these markers were assessed.

**Results**: Forty TNBC histological slides were included, and 23 (57.5%) were Basal-like type TNBC and 17 (42.5%) were Non basal-like type TNBC. Immunoexpression of P63 nucleus and SOX2 was not different between types of TNBC. However, immunoexpression of P63 in the cytoplasm in Basal-like type TNBC was significantly higher than in Non basal-like type TNBC ( *p*=0.021). Predictor diagnostic value analysis suggested that immunoexpression of P63 in cytoplasm had 56.5% sensitivity and 70.6% specificity for diagnosing Basal-like type TNBC, with area under curve of 0.64.

**Conclusions**: Immunoexpression of P63 in the cytoplasm has a relatively weak diagnostic value to discriminate Basal-like and Non basal-like types of TNBC.

## Introduction

Breast cancer, accounting for 25% of all cancer cases and 15% of all cancer deaths among females, is the most frequently diagnosed cancer among female worldwide ^1, 2^. The incidence of breast cancer increased significantly, approximately by 30% in developed countries ^3^ and currently it has been also rising in many developing countries ^2^. In Asia, 639,824 breast cancer cases and 228,926 deaths were recorded in 2012, from which 48,998 cases and 19,750 deaths occurred in Indonesia ^4^. Triple-negative breast cancer (TNBC), a group of breast cancers with the absence of oestrogen receptor and progesterone receptor and no overexpression of human epidermal growth factor receptor 2 (HER2), represents 10%–20% of invasive breast cancer. A global data base, National Cancer Data Base (NCDB), reveals that TNBC was present in 13% of breast cancer patients, ranged from 23.7% in African-Americans to 8.9% in Filipino patients ^5^. In Southeast Asia, a study found that TNBC presented in 10.5% among 1227 breast cancer patients ^6^ . In Indonesia, a study that was conducted between 2010 and 2011 in Bandung found that 11.9% of breast cancer patients were TNBC ^7^.

Immunohistochemically, TNBC could be divided into two subtypes: Basal-like (positive for the expression of high-molecular-weight/basal cytokeratins 5/6 (CK5/6) and epidermal growth factor receptor (EGFR)) and Non basal-like (negative for the expression of CK5/6 and EGFR). Basal-like TNBC usually has p53 mutation, EGFR overexpression, loss of function of BRCA1, c-MYC amplification, and high histological grade indicating more aggressive characteristics and aggressive behavior ^8, 9^. In addition, majority of Basal-like cancer cannot be managed effectively with trastuzumab and hormonal treatments ^10^. 

Advanced screening and diagnosis methods for breast cancer such as mammograms, ultrasound, magnetic resonance imaging and fine-needle aspiration, have allowed for detection of small lesions at the early stage. Identifying breast cancer at the early stage will increase the potential for curative treatment and therefore increases the survival rate ^11– 14^. However, smaller lesions are more challenging to diagnose. Therefore, it is essential to use an advanced immunohistochemical approach for evaluation of smaller tissue specimens that target more specific markers ^15^.

A previous study using a MCF7 breast cancer cell line to produce MCF7-derived tumour xenografts found that P63 and SOX2 immunostainings were two potential markers for breast cancer ^16^. P63, involved in cellular differentiation, is a homolog of tumour protein P53 and in normal breast ducts and lobules it is expressed frequently in the nuclei of myoepithelial cells ^17^. Mutation of the *p53* gene results in a very high risk of breast cancer ^18^. The roles of p63 in tumorigenesis, cancer progression, and metastasis are still being discovered. However, in animal model found that p63 deficiency may be a causative factor for metastatic spread ^19, 20^. In addition, clinical evidence suggests that a robust correlation between reduced p63 expression and cancer progression ^21^.

A study revealed that the total percentage of P63-positive cells was related to marked nuclear pleomorphism and the intensity of P63 staining was associated with syncytial growth pattern in TNBC ^22^. In addition, data also reveals that *p63* gene expression in breast cancer could be used as a specific marker of metaplastic carcinoma ^17^, and P63 immunohistochemical staining could improve diagnostic accuracy of breast cancer even in small tissue specimens ^23^.

SOX2 is a transcription factor belonging to the SOX family and functions as an activator or suppressor of gene transcription ^24, 25^. Data shows that SOX2 promotes cellular proliferation of breast tissue ^26^ and regulates self-renewal in cancer stem cells ^27^. The scientific evidence reveals that SOX2 acts as an oncogene in epithelial cancers ^25^ and in the breast, a study found that silencing of *sox2* gene was associated with reduction of the size of the cancer stem cells and restoration of tamoxifen sensitivity ^28^. All together, these data indicate that P63 and SOX2 have pivotal role in breast cancer and therefore are potential to be used as specific biomarkers. This study was conducted to assess the immunoexpression of P63 and SOX2 in TNBC cases in order to provide insight regarding their potential diagnostic value (single or in combination) to differentiate TNBC types.

## Methods

### Study setting and histological slides

A cross-sectional study to assess the immunoexpression of P63 and SOX2 in TNBC cases (negative expression of estrogen and progesterone receptors and c-erbB2) was conducted. Histological slides of TNBC and their paraffin blocks, tested between the 1 ^st^ of January 2011 and 31 ^st^ of December 2015, were collected from the Pathology Anatomy Laboratory, Dr. Hasan Sadikin Hospital, Bandung, Indonesia. Each histological slide was examined by two certified pathologists. To classify the type of TNBC morphology, between Basal-like type TNBC and Non basal-like type TNBC, cytokeratin 5/6 (CK 5/6) immunohistochemical staining was carried out on all TNBC histological slides. Concurrently, the immunoexpression of P63 and SOX2 was measured using immunohistochemical stains with specific primer antibodies. The protocol of this study was approved by the Health Research Ethical committee of Sumatera Utara University (approval 103/KOMET/FK USU/2015) and the usage of histological specimens was approved by the Pathology Anatomy Laboratory of Dr. Hasan Sadikin Hospital (LB.02.01/B29/239/X/2015).

### Immunohistochemistry

Forty archival paraffin blocks from TNBC cases were subjected to immunohistochemical staining to assess the immunoexpression of CK 5/6, P63 and SOX2. Briefly, 4 μm sections of each paraffin block were prepared using standard procedure ^29^. Immunohistochemical staining was conducted using primary antibodies as follows: anti-CK5/6 monoclonal antibody (Biocare Medical, Concord, CA, USA), anti-P63 monoclonal antibody (Biocare Medical, Concord, CA, USA) and anti-SOX2 monoclonal antibody (Abcam, Cambridge, UK). Starr Trek Universal HRP Detection (Biocare Medical, Concord, CA, USA) was used as second antibody. A chromogen 3,3’-diaminobenzidine (DAB) (Biocare Medical, Concord, CA, USA) was used to develop the colour. For each experiment, appropriate controls were used.

Immunoexpression of CK 5/6 was interpreted as positive or negative, in which positive CK 5/6 indicates Basal-like type TNBC while negative CK 5/6 indicates Non basal-like type TNBC. Immunoexpression of P63 and SOX2 was evaluated using an immunoreactivity scoring system that had been published elsewhere with modification ^22^. Staining intensity was scored as follows: 1 (no staining), 2 (weak staining), 3 (moderate staining) and 4 (strong staining). The percentage of positively stained tumour cells was assessed as a proportion of the total number of tumour cells present in the section as follows: 1 (<20%), 2 (≥20–50%), 3 (>50–80%) and 4 (>80%).

Immunoreactivity score was calculated by multiplying staining intensity and the percentage of positivity, and the score therefore ranged from 1 to 16. The immunoreactive score was then divided into low (≤ 5), moderate (≥ 6 – 10) and high (≥11 – 16). Immunoexpression of P63 was measured both in cytoplasm and nucleus while SOX2 immunoexpression was measured in nucleus only.

### Statistical analysis

Normality of the data was assessed using the Shapiro-Wilk test and therefore the analysis tests chosen based on the normality of the data. The correlations between immunoexpression of P63 (cytoplasm and nucleus) and SOX2 were assessed using Pearson correlation and Spearman correlation, respectively. The associations of P63 and SOX2 immunoexpression and type of TNBC were assessed using Mann Whitney test. The predictive diagnostic values of P63 cytoplasm for diagnosing Basal-like type TNBC were estimated using several immunoreactivity score cut-off points. Receiver operating characteristic curve (ROC) was plotted and area under the ROC curves (AUC) was estimated. For all analyses, estimates were considered statistically significant for two-tailed values of *p*<0.05. All analyses were conducted using Statistical Package for the Social Sciences software (SPSS for Windows, Version 16, Chicago, IL).

## Results

### Clinicopathology and classification of TNBC

The histopathology of the TNBC samples used in this study is described in Table 1. Approximately 45% of the samples were classified as metaplastic carcinomas. In addition, immunohistochemical staining for CK 5/6 revealed that 23 (57.5%) of samples were Basal-like type TNBC and while 17 (42.5%) samples were Non basal-like type TNBC.

**Table 1.** Histopathology types of TNBC samples used in this study (N=40).

Type of histopathology	*n* (%)
Metaplastic carcinoma, spindle cells component	10 (25.0)
Metaplastic carcinoma, producing mucin	3 (7.5)
Metaplastic carcinoma, liposarcoma component	2 (5.0)
Metaplastic carcinoma, squamous cell	1 (2.5)
Metaplastic carcinoma, matrix hyaline	1 (2.5)
Medullary carcinoma	13 (32.5)
Micropapillary carcinoma	1 (2.5)
Invasive ductal carcinoma grade 3	6 (15.0)
Invasive lobular carcinoma grade 3	2 (5.0)
Invasive ductal carcinoma + invasive lobular carcinoma (mixed)	1 (2.5)

### Immunoreactivity score of P63 and SOX2

Immunoexpression of P63 and SOX2 in samples, categorized by immunoreactivity score, are presented in Table 2. For both types of TNBC (basal and non basal-like type), all immunoreactivity scores for P63 in the nucleus were classified as low grade, while 11 (27.5%) and 7 (17.5%) samples were classified as moderate and high grade, respectively for the P63 in the cytoplasm. The immunoreactivity grade for SOX2 was similar to P63 in the cytoplasm, and therefore correlation analyses were conducted.

**Table 2.** Immunoreactivity score of P63 and SOX2 in TNBC.

Grade	P63 cytoplasm, *n* (%)	P63 nucleus, *n* (%)	SOX2, *n* (%)
Low (≤ 5)	22 (55.0)	40 (100.0)	19 (47.5)
Moderate (≥ 6 – 10)	11 (27.5)	0 (0.0)	12 (30.0)
High (≥11 – 16)	7 (17.5)	0 (0.0)	9 (22.5)

### Correlation between immunoexpression of P63 and SOX2

There was a strong negative correlation between immunoexpression of P63 in the cytoplasm and immunoexpression of SOX2 in the nucleus in metaplastic carcinoma (a sub-type of TNBC basal-like type) (r=-0.73, *p=*0.013) ( Table 3). In addition, linear regression showed a relatively strong correlation between P63 cytoplasm and SOX2 immunoexpression in metaplastic carcinoma (r=0.49, *p*=0.012). There was no significant correlation between P63 cytoplasm and SOX2 immunoexpression in Non basal-like type TNBC, and no significant correlation between P63 nucleus and SOX2 immunoexpression either in Basal-like type or Non basal-like type of TNBC.

**Table 3.** Correlation between immunoexpression of P63 cytoplasm and SOX2 in Basal-like type TNBC.

TNBC basal-like type	*n*	P63	SOX2	r	*p*
		Mean (±SD)	Mean (±SD)		
Invasive ductal carcinoma	2	5.50 (3,54)	5.00 (5.66)	-	-
Invasive ductal carcinoma and invasive lobular carcinoma	1	16.00 (0.00)	9.00 (0.00)	-	-
Invasive lobular carcinoma	1	8.00 (0.00)	1.00 (0.00)	-	-
Invasive micropapillary carcinoma	1	12.00 (0.00)	16.00 (0.00)	-	-
Medullary carcinoma	6	5.50 (4.76)	8.00 (5.48)	0.64	0.172
Metaplastic carcinoma	12	6.67 (4.68)	6.00 (3.77)	-0.73	0.013 *

*Significant at 0.05

### Immunoexpression of P63 and SOX2 in Basal-like and Non basal-like type of TNBC

Immunoexpression of P63 cytoplasm, P63 nucleus and SOX2 in Basal-like and Non Basal-like TNBC is shown in Table 4. The data indicates that the immunoexpression of P63 cytoplasm in Basal-like type TNBC was significantly higher compared to Non basal-like type TNBC ( *p*=0.021). Immunoexpression of P63 nucleus and SOX2 was not different between Basal-like and Non basal-like types of TNBC, with *p*-values of *p*=0.27 and *p*=0.17, respectively.

**Table 4.** Immunoexpression of P63 and SOX2 in Basal-like type TNBC and Non basal-like type TNBC.

Marker	TNBC type	*n*	Immunoreactivity score Mean (±SD)	*p*
P63 cytoplasm	Basal-like	23	6.96 (4,73)	0.021 *
	Non basal-like	17	3.76 (4,16)	
P63 nucleus	Basal-like	23	1.22 (0,52)	0.273
	Non basal-like	17	1.06 (0,24)	
SOX2	Basal-like	23	6.78 (4,69)	0.172
	Non basal-like	17	4.82 (3,61)	

*Significant at 0.05

### Predictor diagnostic value of P63 in the cytoplasm for diagnosing Basal like type TNBC

As mentioned above, immunoexpression of P63 in the cytoplasm was the only marker that was significantly different between TNBC types. Therefore, immunoreactivity score of P63 cytoplasm was further analysed to determine its ability to predict Basal-like type TNBC. Table 5 shows the predictive values of P63 in the cytoplasm for determining Basal-like type TNBC, using seven immunoreactivity score cut-off values from 3 to 9. It shows that P63 has relatively weak diagnostic value in diagnosing Basal-like type TNBC. The highest sensitivity was achieved at immunoreactivity score 3, while specificity was increasing with a higher immunoreactivity score.

**Table 5.** Predictor diagnostic value of P63 in the cytoplasm for diagnosing Basal-like type TNBC.

Diagnostic test	Cut-off of P63 cytoplasm immunoreactivity score						
	3	4	5	6	7	8	9
Sensitivity (%)	78.3	73.9	56.5	56.5	52.2	52.2	30.4
Specificity (%)	58.8	58.8	70.6	70.6	82.4	82.4	88.2
Positive predictive value (%)	72.0	70.8	72.2	72.2	80.0	80.0	77.8
Negative predictive value (%)	66.7	62.5	54.5	54.5	56.0	56.0	48.4
Positive likelihood ratio (%)	190.1	179.5	192.2	192.2	295.7	295.7	258.7
Negative likelihood ratio (%)	37.0	44.3	61.6	61.6	58.1	58.1	78.8
Accuracy (%)	70.0	67.5	62.5	62.5	65.0	65.0	55.0

**Figure 1.**
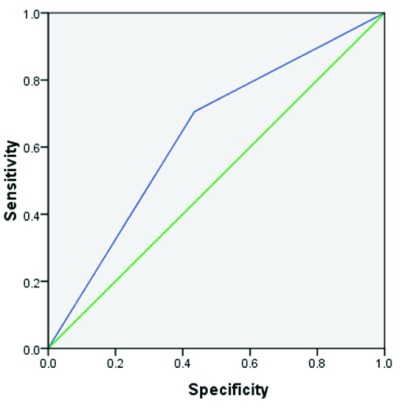
Receiver operating curve of P63 cytoplasm immunoexpression for determining TNBC basal-like type.

Using the average score of P63 cytoplasm immunoexpression for Basal-like type TNBC in this study, 5.6 or 6, the sensitivity and specificity of P63 cytoplasm immunoreactivity score to predict Basal-like type TNBC was 56.5% and 72.6%, respectively with area under curve 0.64. The receiver operating curve of predictive diagnostic value of P63 cytoplasm for determining Basal-like type TNBC is plotted in Figure 1.

Immunoexpression and immunoreactivity scores of P63, SOX2 and CK 5/6 in the forty specimens that were analysed Click here for additional data file. Copyright: © 2018 Kamarlis RK et al. 2018 Data associated with the article are available under the terms of the Creative Commons Zero "No rights reserved" data waiver (CC0 1.0 Public domain dedication).

## Discussion

To the best of our knowledge, this is the first study conducted to assess the immunoexpression of P63 and SOX2 in TNBC cases in Indonesia. Some studies have been conducted to assess the predictive values of P63 as specific marker for breast cancer ^17, 30^. In addition, the idea of utilization of a cocktail of specific markers has been proposed previously to provide higher sensitivity and specificity for diagnosing specific breast cancers ^15, 22, 31^. However, none of the previous studies had been conducted to assess the diagnostic value of immunoexpression of P63 and SOX2 in combination. This study, at the beginning, sought to assess predictive value of combination both of those markers for specific type of TNBC. However, we found that there was no difference in immunoexpression of SOX2 between Basal-like type TNBC and Non basal-like type TNBC. Nevertheless, we found that immunoexpression of P63 cytoplasm, but not P63 nucleus, was higher in Basal-like type TNBC compared to Non basal-like type TNBC.

P63 has been proposed as a breast cancer marker for a long time, but with conflicting results. A study demonstrated that immunoexpression of P63 was associated with breast cancers, for example the metaplastic carcinoma type of breast cancer ^17^, but there was no difference in immunoexpression of P63 between medullary breast carcinomas and atypical medullary breast carcinomas of TNBC ^30^. In our study, we found that the sensitivity and specificity of P63 cytoplasm immunoexpression to diagnose Basal-like type TNBC was 56.5% and 72.6%, respectively, with area under curve of 0.64. This sensitivity and specificity seems higher compared to a previous study, with 14% and 94%, respectively in determining a Basal-like type in infiltrative ductal carcinomas (TNBC) ^22^. All together, these data indicate a weak predictive value of P63 immunoexpression as marker for Basal-like type TNBC. However, a study found that P63 is a specific marker for metaplastic carcinomas of the breast (a sub-type of Basal-like type TNBC) ^17^. In our study, we could not assess the predictive value of P63 cytoplasm immunoexpression for determining metaplastic carcinomas, due to the small sample size (see Table 3).

We found that SOX2 immunoexpression grade was classified as moderate and high grade in 55% of TNBC cases ( Table 2,) and it has been indicated previously that SOX2 has strong roles in promoting breast cancers ^26– 28, 32^. However, there was no different in immunoexpression between Basal-like type TNBC and Non basal-like type TNBC. This result indicates that SOX2 expression is not different amongst TNBC types. This finding was in line with a previous study that indicated that SOX2 was expressed across different breast cancer subtypes ^33^. A study found that SOX2 antibody in the sera was is higher in patients with breast cancer compared to healthy women and therefore it could be used to discriminate between breast cancer patients and healthy controls ^34^. In addition, a meta-analysis found that SOX2 expression was associated with tumor size, histological grade, the aggressiveness and lymph node metastasis in TNBC patients ^35^. All together, these results indicate that there was a possibility SOX2 expression could be used for diagnosing breast cancers, but there was no difference in expression amongst breast cancer types, and therefore it could not be used as specific marker for differentiating TNBC types.

There are some limitations to this study. The sample size was relatively small, and therefore some analyses could not be conducted. In addition, the diagnostic specimens were collected from different procedures such as from biopsy, mastectomy or lumpectomy, and this might influence the immunoexpression of the markers.

## Conclusions

Immunoexpression of P63 cytoplasm is higher among Basal-like type TNBC compared to Non basal-like type TNBC. However, the predictive diagnostic value of P63 immunoexpression in the cytoplasm for Basal-like type TNBC is relatively low, with 56.5% sensitivity and 72.6% specificity.

## Data availability

The data referenced by this article are under copyright with the following copyright statement: Copyright: © 2018 Kamarlis RK et al.

Data associated with the article are available under the terms of the Creative Commons Zero "No rights reserved" data waiver (CC0 1.0 Public domain dedication). 

Dataset 1: Immunoexpression and immunoreactivity scores of P63, SOX2 and CK 5/6 in the forty specimens that were analysed. DOI, 10.5256/f1000research.12671.d179131 ^36^

## References

[1] Key TJ Verkasalo PK Banks E: Epidemiology of breast cancer. *Lancet Oncol.* 2001;2(3):133–140. 10.1016/S1470-2045(00)00254-0 11902563

[2] Torre LA Bray F Siegel RL: Global cancer statistics, 2012. *CA Cancer J Clin.* 2015;65(2):87–108. 10.3322/caac.21262 25651787

[3] Althuis MD Dozier JM Anderson WF: Global trends in breast cancer incidence and mortality 1973–1997. *Int J Epidemiol.* 2005;34(2):405–412. 10.1093/ije/dyh414 15737977

[4] Ghoncheh M Momenimovahed Z Salehiniya H: Epidemiology, Incidence and Mortality of Breast Cancer in Asia. *Asian Pac J Cancer Prev.* 2016;17(S3):47–52. 10.7314/APJCP.2016.17.S3.47 27165207

[5] Plasilova ML Hayse B Killelea BK: Features of triple-negative breast cancer: Analysis of 38,813 cases from the national cancer database. *Medicine (Baltimore).* 2016;95(35):e4614. 10.1097/MD.0000000000004614 27583878 PMC5008562

[6] Alcantara VS Lim GH Lim SH: Incidence and prognosis of non-metastatic triple negative breast cancer (TNBC) among different races in Southeast Asia. *J Surg Oncol.* 2017;115(5):523–537. 10.1002/jso.24559 28168712

[7] Kusumadjayanti N Badudu DF Hernowo BS: Characteristics of patients with estrogen receptor (ER)-negative, progesterone receptor (PR)-negative, and HER2-negative invasive breast cancer in Dr. Hasan Sadikin General Hospital, Bandung, Indonesia from 2010 to 2011. *Althea Med J.* 2015;2(3):391–394. 10.15850/amj.v2n3.494

[8] Cleator S Heller W Coombes RC: Triple-negative breast cancer: therapeutic options. *Lancet Oncol.* 2007;8(3):235–44. 10.1016/S1470-2045(07)70074-8 17329194

[9] Kobayashi S: Basal-like subtype of breast cancer: a review of its unique characteristics and their clinical significance. *Breast Cancer.* 2008;15(2):153–8. 10.1007/s12282-008-0034-3 18311481

[10] Rakha EA Reis-Filho JS Ellis IO: Basal-like breast cancer: a critical review. *J Clin Oncol.* 2008;26(15):2568–2581. 10.1200/JCO.2007.13.1748 18487574

[11] AIHW: Breast cancer survival by size and nodal status in Australia.In: Registries NBCCAAoC, ed. *Cancer series no. 39* Canberra: AIHW;2007 Reference Source

[12] Allemani C Minicozzi P Berrino F: Predictions of survival up to 10 years after diagnosis for European women with breast cancer in 2000–2002. *Int J Cancer.* 2013;132(10):2404–2412. 10.1002/ijc.27895 23047687

[13] Allemani C Weir HK Carreira H: Global surveillance of cancer survival 1995–2009: analysis of individual data for 25,676,887 patients from 279 population-based registries in 67 countries (CONCORD-2). *Lancet.* 2015;385(9972):977–1010. 10.1016/S0140-6736(14)62038-9 25467588 PMC4588097

[14] Naroda S Iqbala J Miller AB: Why have breast cancer mortality rates declined? *J Cancer Policy.* 2015;5:8–17. 10.1016/j.jcpo.2015.03.002

[15] Reisenbichler ES Ross JR Hameed O: The clinical use of a P63/cytokeratin7/18/cytokeratin5/14 antibody cocktail in diagnostic breast pathology. *Ann Diagn Pathol.* 2014;18(6):313–318. 10.1016/j.anndiagpath.2014.08.007 25224390

[16] Liu Y Coates PJ Nenutil R: Lack of correlation between markers of breast cancer initiating cells. *Breast Cancer Res.* 2010;12(Suppl 1):P38 10.1186/bcr2535

[17] Koker MM Kleer CG: p63 expression in breast cancer: a highly sensitive and specific marker of metaplastic carcinoma. *Am J Surg Pathol.* 2004;28(11):1506–1512. 10.1097/01.pas.0000138183.97366.fd 15489655

[18] Assi HA Khoury KE Dbouk H: Epidemiology and prognosis of breast cancer in young women. *J Thorac Dis.* 2013;5 Suppl 1:S2–8. 10.3978/j.issn.2072-1439.2013.05.24 23819024 PMC3695538

[19] Flores ER Sengupta S Miller JB: Tumor predisposition in mice mutant for *p63* and *p73*: evidence for broader tumor suppressor functions for the *p53* family. *Cancer Cell.* 2005;7(4):363–373. 10.1016/j.ccr.2005.02.019 15837625

[20] Su X Chakravarti D Cho MS: *TAp63* suppresses metastasis through coordinate regulation of *Dicer* and miRNAs. *Nature.* 2010;467(7318):986–990. 10.1038/nature09459 20962848 PMC3055799

[21] Bergholz J Xiao ZX: Role of p63 in Development, Tumorigenesis and Cancer Progression. *Cancer Microenviron.* 2012;5(3):311–322. 10.1007/s12307-012-0116-9 22847008 PMC3460051

[22] Thike AA Cheok PY Jara-Lazaro AR: Triple-negative breast cancer: clinicopathological characteristics and relationship with basal-like breast cancer. *Mod Pathol.* 2010;23(1):123–133. 10.1038/modpathol.2009.145 19855377

[23] Harton AM Wang HH Schnitt SJ: p63 Immunocytochemistry improves accuracy of diagnosis with fine-needle aspiration of the breast. *Am J Clin Pathol.* 2007;128(1):80–85. 10.1309/RX1W80K68NRJ0PTT 17580273

[24] Weina K Utikal J: SOX2 and cancer: current research and its implications in the clinic. *Clin Transl Med.* 2014;3:19. 10.1186/2001-1326-3-19 25114775 PMC4126816

[25] Sarkar A Hochedlinger K: The sox family of transcription factors: versatile regulators of stem and progenitor cell fate. *Cell Stem Cell.* 2013;12(1):15–30. 10.1016/j.stem.2012.12.007 23290134 PMC3608206

[26] Stolzenburg S Rots MG Beltran AS: Targeted silencing of the oncogenic transcription factor *SOX2* in breast cancer. *Nucleic Acids Res.* 2012;40(14):6725–6740. 10.1093/nar/gks360 22561374 PMC3413152

[27] Leis O Eguiara A Lopez-Arribillaga E: Sox2 expression in breast tumours and activation in breast cancer stem cells. *Oncogene.* 2012;31(11):1354–1365. 10.1038/onc.2011.338 21822303

[28] Piva M Domenici G Iriondo O: Sox2 promotes tamoxifen resistance in breast cancer cells. *EMBO Mol Med.* 2014;6(1):66–79. 10.1002/emmm.201303411 24178749 PMC3936493

[29] Anderson G Bancroft J: Tissue processing and microtomy.In: Bancroft JG, M., ed. *Theory and practiceal of histological techniques 5th Edition.*Edinburgh Churchill Livingstone;2002;109–123.

[30] Matkovic B Juretic A Separovic V: Immunohistochemical analysis of ER, PR, HER-2, CK 5/6, p63 and EGFR antigen expression in medullary breast cancer. *Tumori.* 2008;94(6):838–844. 19267102 10.1177/030089160809400611

[31] Tacha DE Bloom K Kyshtoobayava A: A double immunostaining technique with a cocktail CK5, CK14, p63, CK7 and CK18 distinguishes between hyperplasia of the usual type, atypical hyperplasia, microinvasive and basal phenotype breast cancers. *Modern Pathology.* 2009;22:388a.

[32] Liu K Xie F Gao A: SOX2 regulates multiple malignant processes of breast cancer development through the SOX2/miR-181a-5p, miR-30e-5p/TUSC3 axis. *Mol Cancer.* 2017;16(1):62. 10.1186/s12943-017-0632-9 28288641 PMC5348847

[33] Lengerke C Fehm T Kurth R: Expression of the embryonic stem cell marker SOX2 in early-stage breast carcinoma. *BMC Cancer.* 2011;11:42. 10.1186/1471-2407-11-42 21276239 PMC3038979

[34] Sun Y Zhang R Wang MJ: SOX2 autoantibodies as noninvasive serum biomarker for breast carcinoma. *Cancer Epidemiol Biomarkers Prev.* 2012;21(11):2043–2047. 10.1158/1055-9965.EPI-12-0498 22832207

[35] Zheng Y Qin B Li F: Clinicopathological significance of Sox2 expression in patients with breast cancer: a meta-analysis. *Int J Clin Exp Med.* 2015;8(12):22382–22392. 26885218 PMC4730004

[36] Reno KK Muhammad NDL: Dataset 1 in: Immunoexpression of P63 and SOX2 in triple-negative breast cancers, Indonesia. *F1000Research.* 2017 Data Source 10.12688/f1000research.12671.1 PMC5820604 29527291

