# Supplementary material for: Immunoexpression of P63 and SOX2 in triple-negative breast cancers, Indonesia
Source: F1000Res. 2018 Jan 8;6:1780. Originally published 2017 Sep 28. [Version 2] doi: 10.12688/f1000research.12671.2 (PMC5820604; doi:10.12688/f1000research.12671.2)
Supplement: Peer review history

# Peer review: Referee response for version 2

**DOI:** 10.5256/f1000research.14420.r29568

**Published:** 2018-02-20

**Authors:** Diah Rini Handjari

**Affiliations:** 1 University of Indonesia, Jakarta, Indonesia

This revised version is good and sufficient for indexing.

I have read this submission. I believe that I have an appropriate level of expertise to confirm that it is of an acceptable scientific standard.

# Peer review: Referee response for version 2

**DOI:** 10.5256/f1000research.14420.r29569

**Published:** 2018-02-06

**Authors:** Irianiwati Widodo

**Affiliations:** 1 Department of Anatomical Pathology, Faculty of Medicine, Gadjah Mada University, Yogyakarta, Indonesia

This revised version is good and already sufficient for publication.

I have read this submission. I believe that I have an appropriate level of expertise to confirm that it is of an acceptable scientific standard.

# Peer review: Referee response for version 1

**DOI:** 10.5256/f1000research.13722.r26782

**Published:** 2017-10-20

**Authors:** Diah Rini Handjari

**Affiliations:** 1 University of Indonesia, Jakarta, Indonesia

I. - In introduction there is only little information regarding of nuclear expression of p63.

    - It doesn't mention about cytoplasmic p63 expression.

      What is significance function of p63 in tumor progression or disease progression?

      It written in result and analysis.

    - In introduction you haven't discussed yet about the incidence of triple negative breast cancer in the worldwide, Asia or Indonesia ?

 II. In the methods, to classify TNBC morphology into Basal like and non Basal like, based on only, antibody (CK 5/6) but you didn't perform EGFR staining. Which is one of the marker of TNBC.  

     - Is there any reference about the scoring of intensity and the percentage of positivity of expression of p63?

     - Is there any reference about imunoreactive score?

 III. In discussion, there is also no information about cytoplasmic p63 and its role in disease progression.

     - Researcher only stated that cytoplasmic p63 expression has predictive value to classify breast cancer into TNBC and there is no comparison of p63 expression sensitivity and specificity with other TNBC marker as like CK 5/6 and EGFR which has been routinely used.

I have read this submission. I believe that I have an appropriate level of expertise to confirm that it is of an acceptable scientific standard, however I have significant reservations, as outlined above.

# Response

**Published:** 2017-11-25

**Authors:** Harapan Harapan

**Affiliations:** Syiah Kuala University, Indonesia

Thank you for comments and suggestion. We have deleted some sentences related to the general introduction of breast cancer and added specific information related to TNBC. We would like to confirm that predictive diagnostic analysis in this study was conducted using qualified statistician. There is no evidence to support the importance of the negative correlation between immunoexpression of P63 cytoplasm and immunoexpression SOX2 nucleus in metaplastic carcinoma, and therefore we are unable to discuss this finding in depth. In conclusion section, we have added some additional principal finding as suggested by the reviewer.

# Response

**Published:** 2017-11-25

**Authors:** Harapan Harapan

**Affiliations:** Syiah Kuala University, Indonesia

Thank you for comments and suggestion. We have deleted some sentences related to the general introduction of breast cancer and added specific information related to TNBC. In the revised manuscript, the data of the incidence from global, Southeast Asian countries and Indonesia are included.

In our study, we used the histological paraffin blocks that have been confirmed as TNBC previously by testing ER, PR and HER2. To differentiate Basal like and non Basal like, either CK 5/6 or EGFR staining could be used with no significant different sensitivity and specificity (Livasy *et al.*, 2006; Nielsen *et al.*, 2004). In this study, CK 5/6 was employed to differentiate TNB into Basal like and non Basal like and this procedure was conducted during the study. This method is established method to define basal phenotype (Sasa *et al.*, 2008; Rakha *et al.*, 2007).

In our study, we used the histological paraffin blocks that have been confirmed as TNBC previously by testing ER, PR and HER2. To differentiate Basal like and non Basal like, either CK 5/6 or EGFR staining could be used with no significant different sensitivity and specificity (Livasy *et al.*, 2006; Nielsen *et al.*, 2004). In this study, CK 5/6 was employed to differentiate TNB into Basal like and non Basal like and this procedure was conducted during the study. This method is established method to define basal phenotype (Sasa *et al.*, 2008; Rakha *et al.*, 2007). CK 5/6 and EGFR.

As mentioned Immunohistochemistry Section, the principle of the scoring system for immunoexpression of P63 and SOX2 used in this study was have been elsewhere with modification (Thike *et al.*, 2010). In detailed: Staining intensity was scored as follows: 1 (no staining), 2 (weak staining), 3 (moderate staining) and 4 (strong staining). The percentage of positively stained tumour cells was assessed as a proportion of the total number of tumour cells present in the section as follows: 1 (<20%), 2 (≥20–50%), 3 (>50–80%) and 4 (>80%). Then from staining intensity and percentage of positively stained cells, we created immunoreactivity score by multiplying staining intensity and the percentage of positivity. The score, therefore, ranged from 1 to 16 (divided into low (≤ 5), moderate (≥ 6 – 10) and high (≥11 – 16). However, specifications for staining intensity and the percentage of positivity used in this study are the standard system used by Pathology Anatomy Laboratory of Dr. Hasan Sadikin Hospital Bandung since 1990. These score systems are also adopted in some breast cancer diagnostic centres in Indonesia.

Immunoreactive score system used in this study have been published elsewhere (Thike *et al.*, 2010). We mentioned this in Immunohistochemistry Section of our Methods.

In this study produce all raw data related to: type of TNBC (Basal like and Non Basal-like), subtype of cancer, expression of SOX2, P63 and CK 5/6 including staining intensity, distribution of the positively cells and immunoreactive score.

Reference:

Thike AA, Cheok PY, Jara-Lazaro AR, et al. Triple-negative breast cancer: clinicopathological characteristics and relationship with basal-like breast cancer. Mod Pathol. 2010;23(1):123–133.

Livasy CA, Karaca G, Nanda R, et al. Phenotypic evaluation of the basal-like subtype of invasive breast carcinoma. Mod Pathol 2006;19:264-271

Nielsen TO, Hsu FD, Jensen K, et al. Immunohistochemical and clinical characterization of the basal-like subtype of invasive breast carcinoma Clin Cancer Res, 2004;10:5367-5374

Rakha EA, El-Sayed ME, Green AR, et al. Breast carcinoma with basal differentiation: a proposal for pathology definition based on basal cytokeratin expression. Histopathology. 2007 Mar;50(4):434-8.

Sasa M, Bando Y, Takahashi M, et al. Screening for basal marker expression is necessary for decision of therapeutic strategy for triple-negative breast cancer. J Surg Oncol, 2008; 97: 30-34

# Peer review: Referee response for version 1

**DOI:** 10.5256/f1000research.13722.r26921

**Published:** 2017-10-12

**Authors:** Irianiwati Widodo

**Affiliations:** 1 Department of Anatomical Pathology, Faculty of Medicine, Gadjah Mada University, Yogyakarta, Indonesia

**Introduction: **

- Do not explain the epidemiology of breast cancer too long, but explain more detail about TNBC and its subtypes (Basal-like and non basal-like), such as differences in histological type, behaviour, prognosis, treatment etc. So, the role of p63 and SOX2 on TNBC becomes clearer.

**Method:**

- Statistical analysis predictive diagnostic value of cytoplasmic p63 expression of basal-like and non basal-like TNBC, as far as I know there should be also negative p63 expression. Therefore I suggest to ask a qualified statistician.

**Discussion:**

- Explain the importance of negative correlation between p63 expression and metaplastic carcinoma. Compare with previous studies.

**Conclusion:**

 Mention also other important results of this study such as:

- The frequency of non basal-like is higher than basal-like.
- The most common histological type of TNBC is metaplastic carcinoma.
- Negative correlation between p63 expression and metaplastic carcinoma
- SOX2 expression in TNBC, even there is no stattistically significant

I have read this submission. I believe that I have an appropriate level of expertise to confirm that it is of an acceptable scientific standard, however I have significant reservations, as outlined above.
